# Protein Elicitor EsxA Induces Resistance to Seedling Blight and PR Genes Differential Transcription in Rice

**DOI:** 10.1186/s12284-021-00532-6

**Published:** 2021-11-04

**Authors:** Wen Qing Yu, Peng Li, Feng Chao Yan, Gui Ping Zheng, Wen Zhi Liu, Wen Xi Lin, Yi Wang, Zhi Qing Luo

**Affiliations:** 1 College of Life Sciences, Shangrao Normal University, Shanrao, 334001 Jiangxi China; 2 Heilongjiang Academy of Land Reclamation Sciences, Harbin, 150038 China; 3 Heilongjiang Bayi Agricultural University, Daqing, 163319 Heilongjiang China

**Keywords:** Protein elicitor, EsxA, Induced systemic resistance, Transcriptome, Pathogenesis-related protein, Signal transduction

## Abstract

Protein elicitors can induce plant systemic resistance to pathogens. In an earlier study, we cloned an EsxA gene from the plant growth-promoting rhizobacterium *Paenibacillus terrae* NK3-4 and expressed it in *Pichia pastoris*. In addition to being important for the pathogenicity of animal pathogens, EsxA can also induce an immune response in animals. While, we found the exogenously expressed EsxA has the activity of elicitor, which can trigger hypersensitive response and reactive oxygen species burst in leaves as well as enhanced rice plant growth. The effects of EsxA on seedling blight (*Fusarium oxysporum*) resistance and gene transcription, including pathogenesis-related (PR) genes in rice were evaluated. The germination rate was 95.0% for seeds treated with EsxA and then inoculated with *F. oxysporum*, which was 2.8-times higher than that of *F. oxysporum*-infected control seeds that were not treated with EsxA (Con). The buds and roots of EsxA-treated seedlings were 2.4- and 15.9-times longer than those of Con seedlings. The plants and roots of seedlings dipped in an EsxA solution and then inoculated with *F. oxysporum* were longer than those of the Con seedlings. Theplant length, number of total roots, and number of white roots were respectively 23.2%, 1.74-times, and 7.42-times greater for the seedlings sprayed with EsxA and then inoculated with *F. oxysporum* than for the Con seedlings. The EsxA induction efficiency (spray treatment) on seedling blight resistance was 60.9%. The transcriptome analysis revealed 1137 and 239 rice genes with EsxA-induced up-regulated and down-regulated transcription levels, respectively. At 48 h after the EsxA treatment, the transcription of 611 and 160 genes was up-regulated and down-regulated, respectively, compared with the transcription levels for the untreated control at the same time-point. Many disease resistance-related PR genes had up-regulated transcription levels. The qPCR data were consistent with the transcriptome sequencing results. EsxA triggered rice ISR to seedling blight and gene differential transcription, including the up-regulated transcription of rice PR genes. These findings may be relevant for the use of EsxA as a protein elicitor to control plant diseases.

**Supplementary Information:**

The online version contains supplementary material available at 10.1186/s12284-021-00532-6.

## Background

Elicitors can induce plant resistance via signal recognition, signal transduction, and defense gene regulation. Protein elicitors are the most important type of elicitors that activate host plant defense responses (Shen et al. 2019) to enhance disease resistance (Ruiz et al. 2018; Mao et al. 2010; Wang et al. 2012; Qiu et al. 2009) and insect resistance (Li et al. 2020). They also stimulate plant metabolism to regulate plant growth (Darwati et al. 2018). Recent research on the mechanism underlying elicitor-induced plant stress resistance revealed that pathogenesis-related (PR) proteins may be elicitor receptors that directly or indirectly bind to elicitors, after which they activate a series of downstream defense-related signal transduction pathways, and trigger plant broad-spectrum induced systemic resistance (ISR) to diseases (Liu et al. 2018; Li et al. 2019).

Early secreted antigenic target of 6 kDa (ESAT-6) is encoded by the EsxA gene, which belongs to the WXG super family. This protein, which was identified in animal pathogens (Pollock and Andersen 1997; Schulthess et al. 2012; Ma et al. 2015), functions in the Type VII secretion system. It has been studied as a virulence factor for bacterial pathogens, and its importance for pathogenicity has been confirmed (Berthet et al. 1998; Ulrichs et al. 1998). However, EsxA is not a simple virulence factor. In addition to being important for the pathogenicity of animal pathogens, it can also induce an immune response in animals (Zhou et al. 2013).

In an earlier study, we cloned an EsxA gene from the plant growth-promoting rhizobacterium *Paenibacillus terrae* NK3-4 and expressed it in *Pichia pastoris*, with the secreted protein inducing a hypersensitive response (HR) and reactive oxygen species (ROS) burst in leaves as well as enhanced rice plant growth (Yu et al. 2021). To further assess whether EsxA can induce plant disease resistance, we used various methods to treat rice with EsxA to evaluate its effect on rice resistance to seedling blight. Transcriptome sequencing and quantitative real-time PCR (qPCR) analyses were combined to investigate the transcription of rice genes, including PR genes, to clarify the mechanism underlying EsxA-induced plant disease resistance at the transcriptional level. The resulting data may form the basis of future research on the role of receptor proteins in cells during interactions between EsxA and plants.

## Results

### EsxA Induction Effect on Rice Seedling Blight Resistance

#### EsxA Induction Effect After the Seed-Dipping Treatment

**Fig. 1:**
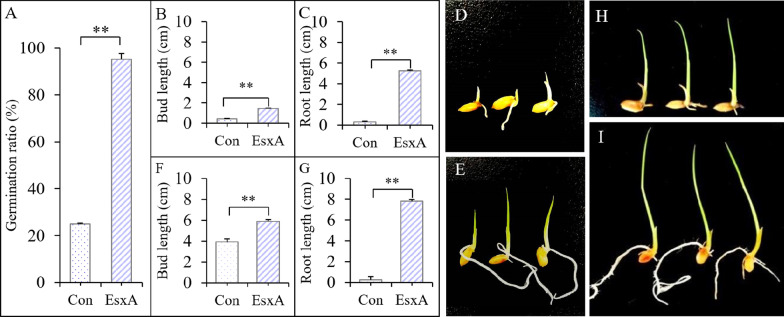
Effects of protein elicitor EsxA on the germination and growth of rice under seedling blight (*Fusarium oxysporum*) stress (seed-dipping treatment). Bar diagrams **A**, **B**, **C**, **D** and **E** present the results at 4 days after rice was challenged with *Fusarium oxysporum*; **F**, **G**, **H** and **I** represent 8 days after rice was challenged with *F. oxysporum*, respectively, with “Con” representing the control treatment (without EsxA) and “EsxA” representing the EsxA treatment (**P < 0.01)

At 4 days after the inoculation with the *F. oxysporum* spore suspension, only 25.0% of the Con seeds (i.e., not treated with EsxA) germinated, whereas 95.0% of the EsxA-treated seeds germinated, with a germination rate 2.8-times higher than that of the Con seeds (*F* = 1622.49, *P* < 0.001) (Fig. 1A). The bud length and the root length of the EsxA-treated rice were 2.4-times (*F* = 4800.50, *P* < 0.001) (Fig. 1B; D compared with E) and 15.9-times (*F* = 1095.200, *P* < 0.001) (Fig. 1 C; D compared with E) greater than the corresponding lengths of the Con rice, respectively. After another 4-day incubation, no newly germinated seed in both treatment, while the EsxA rice bud length was longer than Con (*F* = 88.506, *P* = 0.001) (Fig. 1F; H compared with I). while the Con rice exhibited obvious seedling blight symptoms, with retarded bud and root growth and yellowish-brown decaying roots (Fig. 1 H), because of the roots roted, the root length shorter than that before 4 days (Fig. 1, C comared with G of Con; H compared with D). In contrast, the roots of EsxA-treated rice continued to grow, and remained white (Fig. 1I), meanwhile, the root length of EsxA rice was longer than that of Con rice (*F* = 821.884, *P* < 0.001) (Fig. 1G; H compared with I). Thus, the seed-dipping EsxA treatment promoted the growth of rice seedlings infected by *F. oxysporum*, with minimal disease symptoms. At 4th day after infected by *F. oxysporum*, the induction efficiency for the germination rate, bud length, and root length was 280.7%, 238.5%, and 493.3%, respectively. And the induction efficiency for the bud length and root length was 50.9% and 2879.6% at 8 days infected by *F. oxysporum*, respectively.

#### EsxA Induction Effect After the Seedling-Dipping Treatment

**Fig. 2:**
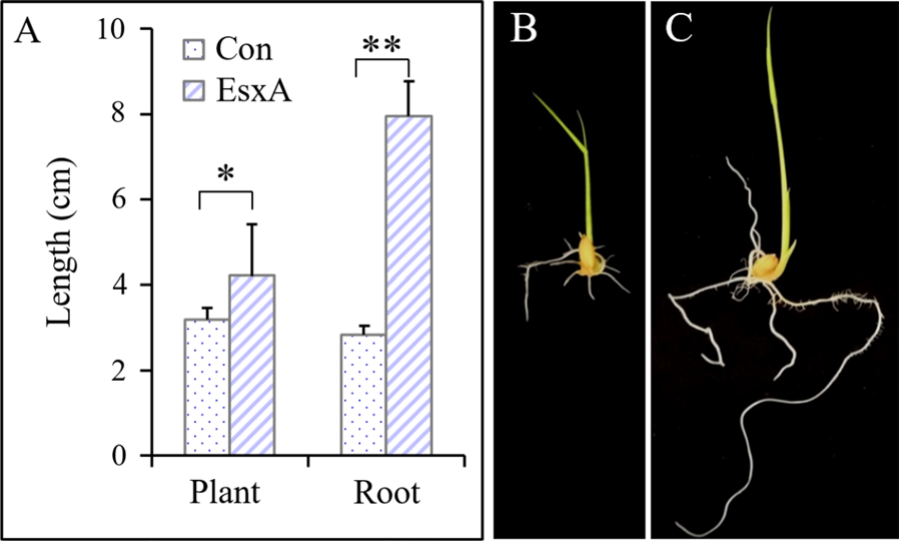
Effects of protein elicitor EsxA on the plant and root length of rice under seedling blight (*Fusarium oxysporum*) stress (seedling-dipping treatment). Bar diagram **A** presents the results at 5 days after rice was challenged with *Fusarium oxysporum*, with “Con” representing the control treatment (without EsxA) and “EsxA” representing the EsxA treatment; **B** and **C** represent “Con” and “EsxA” respectively (*P < 0.05, **P < 0.01))

After a 48-h treatment with EsxA (100 μg/mL) and a 5-day incubation following an inoculation with *F. oxysporum*, the bud length and root length of the EsxA-treated rice were respectively 32.1% (*F* = 17.344, *P* = 0.014) and 1.8-times (*F* = 24.324, *P* = 0.008) greater than the corresponding lengths of the Con rice (Fig. 2A; compared B with C). The induction efficiency for the bud length and root length was 32.1% and 179.8%, respectively. These results indicated that the seedling-dipping treatment with EsxA promoted the growth of rice seedlings infected with *F. oxysporum*.

#### EsxA Induction Effect After the Seedling-Spraying Treatment

**Fig. 3:**
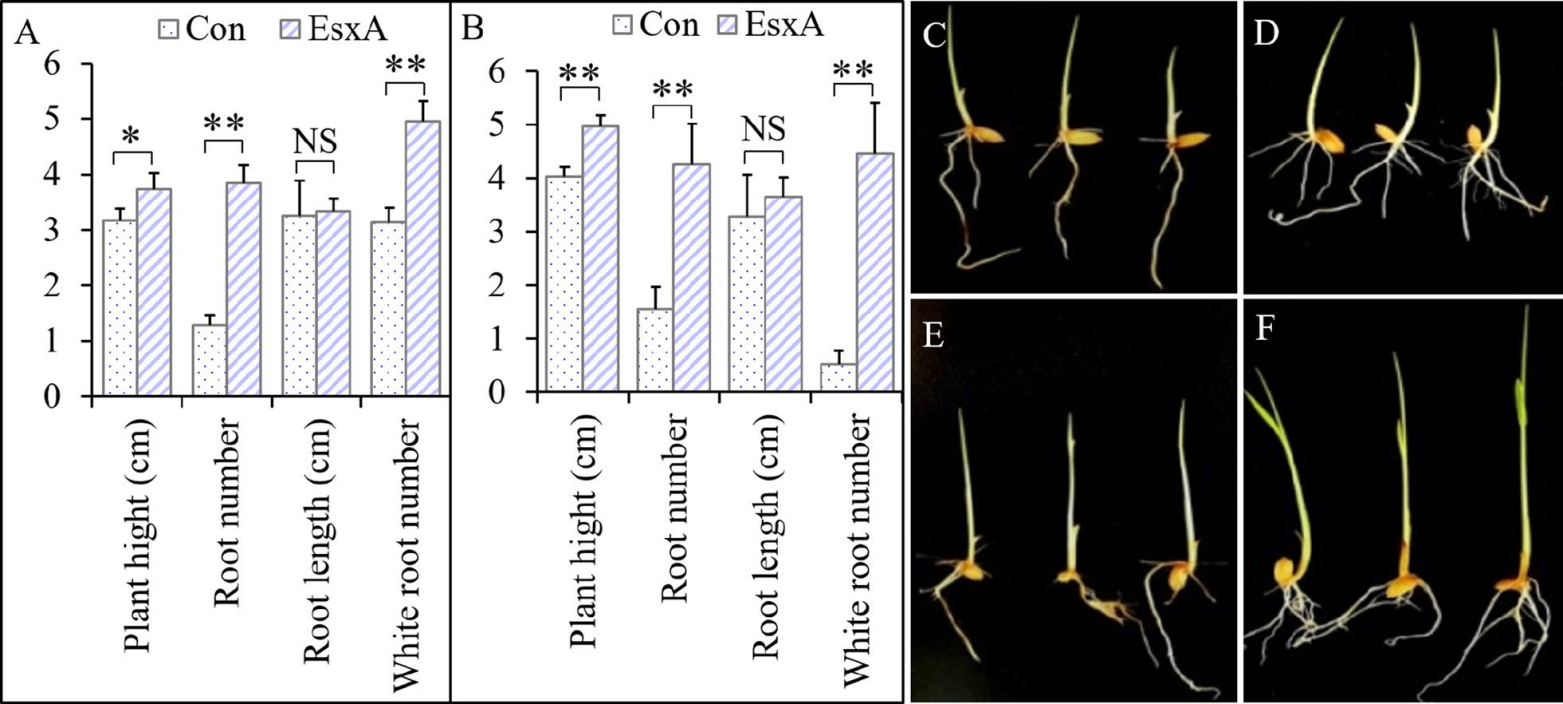
Effects of protein elicitor EsxA on the growth and performances of rice udner seedling blight (*Fusarium oxysporum*) stress (seedling-spraying treatment, 7 days). **A**, **C**, and **D**: 48 h after the EsxA treatment; **B**, **E**, and **F**: 7 days after the inoculation with *Fusarium oxysporum*, with **C** and **D** representing control (Con) seedlings (without EsxA) and **E** and **F** representing seedlings sprayed with EsxA. The number of roots was determined based on roots longer than 1 cm; the number of white roots includes roots shorter than 1 cm (*P < 0.05, **P < 0.01)

At 48 h after the EsxA treatment, the plant height, number of roots, and number of white roots were respectively 17.5% (*F* = 9.491, *P* = 0.015), 2.0-times (*F* = 192.753, *P* < 0.001), and 58.0% (*F* = 68.016, *P* < 0.001) greater for the EsxA-treated rice than for the Con rice. The EsxA treatment had no significant effect on root length (Fig. 3A; C compared with D). At 7 days after the inoculation with *F. oxysporum*, the plant height, number of roots, and number of white roots were respectively 23.2% (*F* = 48.790, *P* < 0.001), 1.74-times (*F* = 38.018, *P* < 0.001), and 7.42-times (*F* = 63.888, *P* < 0.001) greater for the EsxA-treated rice than for the Con rice (Fig. 3B; E compared with F). The results implied that the EsxA treatment alleviated the adverse effects of *F. oxysporum* on rice growth.

At 7 days after the inoculation with *F. oxysporum*, the Con roots were yellow and the number of white roots decreased by 83.1% compared with that before the inoculation (*F* = 214.554, *P* < 0.001). In contrast, there was no significant decrease in the number of white roots for the EsxA-treated rice inoculated with *F. oxysporum* (*F* = 0.966, *P* = 0.354) (Fig. 3). These results indicated that the EsxA spray-treatment promoted rice seedling growth and development, while also significantly decreasing the detrimental effects of *F. oxysporum* on seedling growth.

**Fig. 4:**
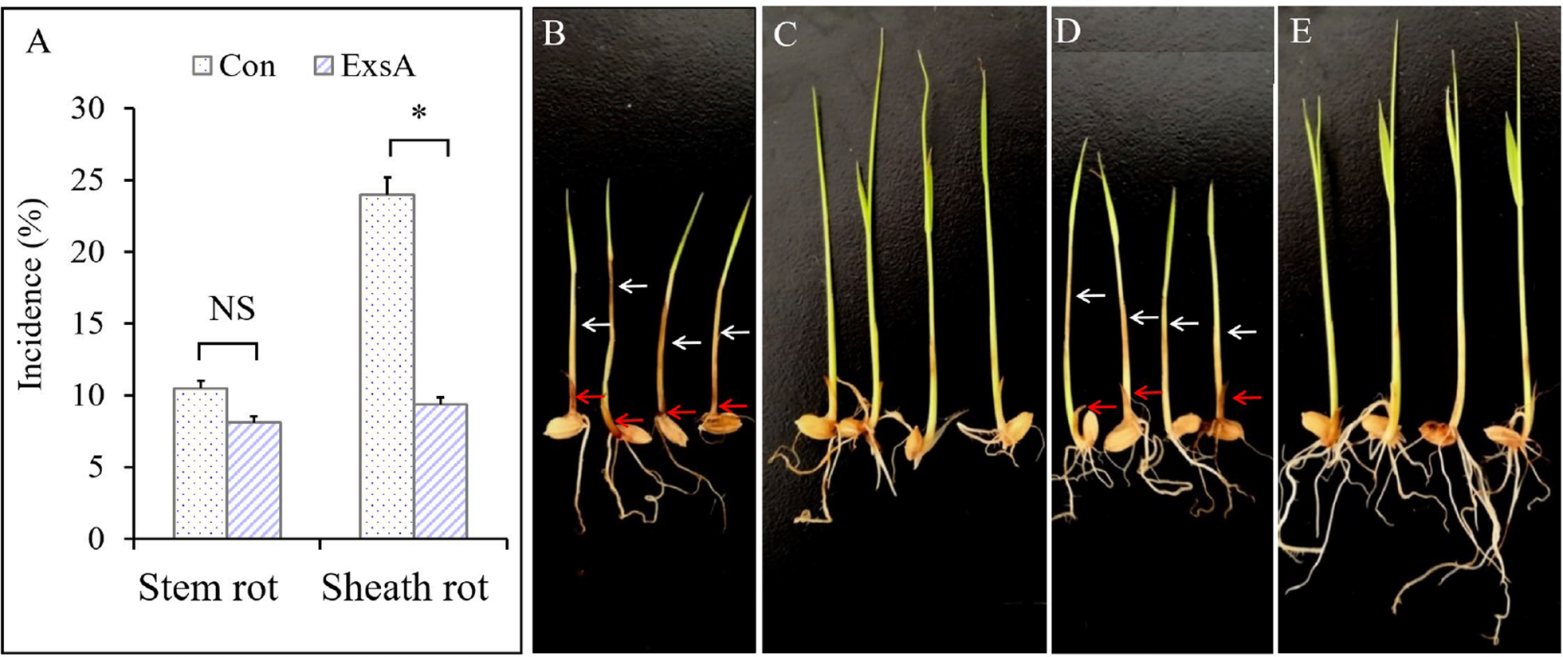
Effects of protein elicitor EsxA on the incidence of rice seedling blight (seedling-spraying treatment, 14 days). **A** representing the incidence of stem rot and sheath rot respectively, **B** and **C**: control (Con) seedlings (sprayed with bovine serum albumin), **D** and **E**: EsxA-treated plants, with **B** and **D** representing the plants exhibiting stem rot (white arrows) and sheath rot (red arrows) symptoms; **C** and **E** representing the plants exhibiting sheath rot symptoms (*P < 0.05; NS: not significant)

At 14 days after the inoculation with *F. oxysporum*, the seedling blight incidence of EsxA-treated rice was significantly lower than that of Con rice. More specifically, the incidence of sheath rot and stem rot among the Con plants was 10.5% and 24.0%, respectively, for a seedling blight incidence of 34.5%. The incidence of sheath rot and stem rot among the EsxA-treated plants was 8.1% and 9.4%, respectively, for a seedling blight incidence of 17.5%. The EsxA treatment significantly decreased the incidence of stem rot (*F* = 5.400, *P* = 0.032) and the overall seedling blight incidence (*F* = 8.455, *P* = 0.009). The EsxA induction efficiency on rice sheath rot and stem rot resistance was 13.8% and 60.9%, respectively (Fig. 4A). The EsxA treatment decreased the incidence and severity of rice seedling blight (Fig. 4, B compared with D). Additionally, the roots of EsxA-treated rice grew significantly better than the Con roots, with whiter roots, longer roots, and more roots (Fig. 4, C compared with E). Furthermore, the Con roots produced a strong odor similar to rotting pear, which is a hallmark characteristic of seedling blight, whereas the roots of the EsxA-treated rice were odorless.

### Effects of EsxA on the Rice Transcriptome

#### Effects of EsxA on Gene Transcription in Rice

**Fig. 5:**
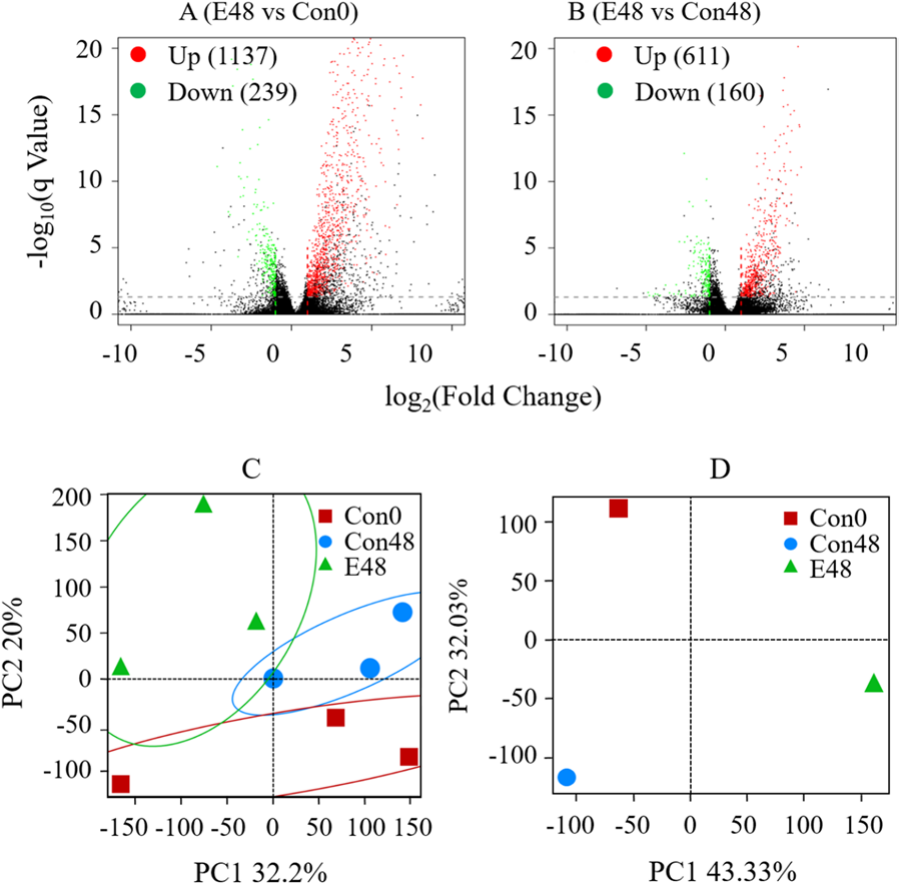
Volcano map (**A**, **B**) and principal coordinate analysis (**C**, **D**) of protein elicitor EsxA-induced rice gene transcription in the compared sample groups. E48: EsxA treatment after 48 h of EsxA treating rice; Con0: control without EsxA at 0 h of EsxA treating rice; Con48: control without EsxA at 48 h after EsxA treating rice. Principal coordinate was conducted using Biological replicates (**C**) and groupmerge (**D**)

The rice transcriptome was sequenced at the two-leaf stage. Specifically, the following samples were analyzed: rice before the EsxA treatment (Con0), rice at 48 h after the EsxA treatment (E48), and control rice (without EsxA treatment) at 48 h (Con48). The RNA-seq raw data of nine samples (three biological replicates in each treatment) were submitted to NCBI bioSample database (accessions: SAMN19404905–SAMN19404913). A total of 36,844 genes and 43,397 transcripts were detected in the rice transcriptome library, including 14,029 new transcripts. A comparison between Con0 and E48 revealed 1376 differentially transcribed genes (i.e., transcription levels differed by more than 2 times, the same belows), of which 1137 were up-regulated and 239 were down-regulated in the EsxA-treated rice (Fig. 5A). A comparison between E48 and Con48 detected 771 differentially transcribed genes, of which 611 were up-regulated and 160 were down-regulated in the EsxA-treated rice (Fig. 5B). Thus, EsxA modulated the transcription of many rice genes, with substantially more up-regulated genes than down-regulated genes.

The principal componet analysis of rice gene transcription indicated that E48 was displaced along the PC1 axes when compared with Con0 and Con48 (Fig. 5C, D). Additionally, Con48 and E48 were displaced along the PC2 axis when compared with Con0. The three EsxA treatment groups formed three spatially clusters. These results suggested that rice gene transcription induced by EsxA.

#### Functional Analysis of EsxA-induced Differentially Transcribed Genes

**Fig. 6:**
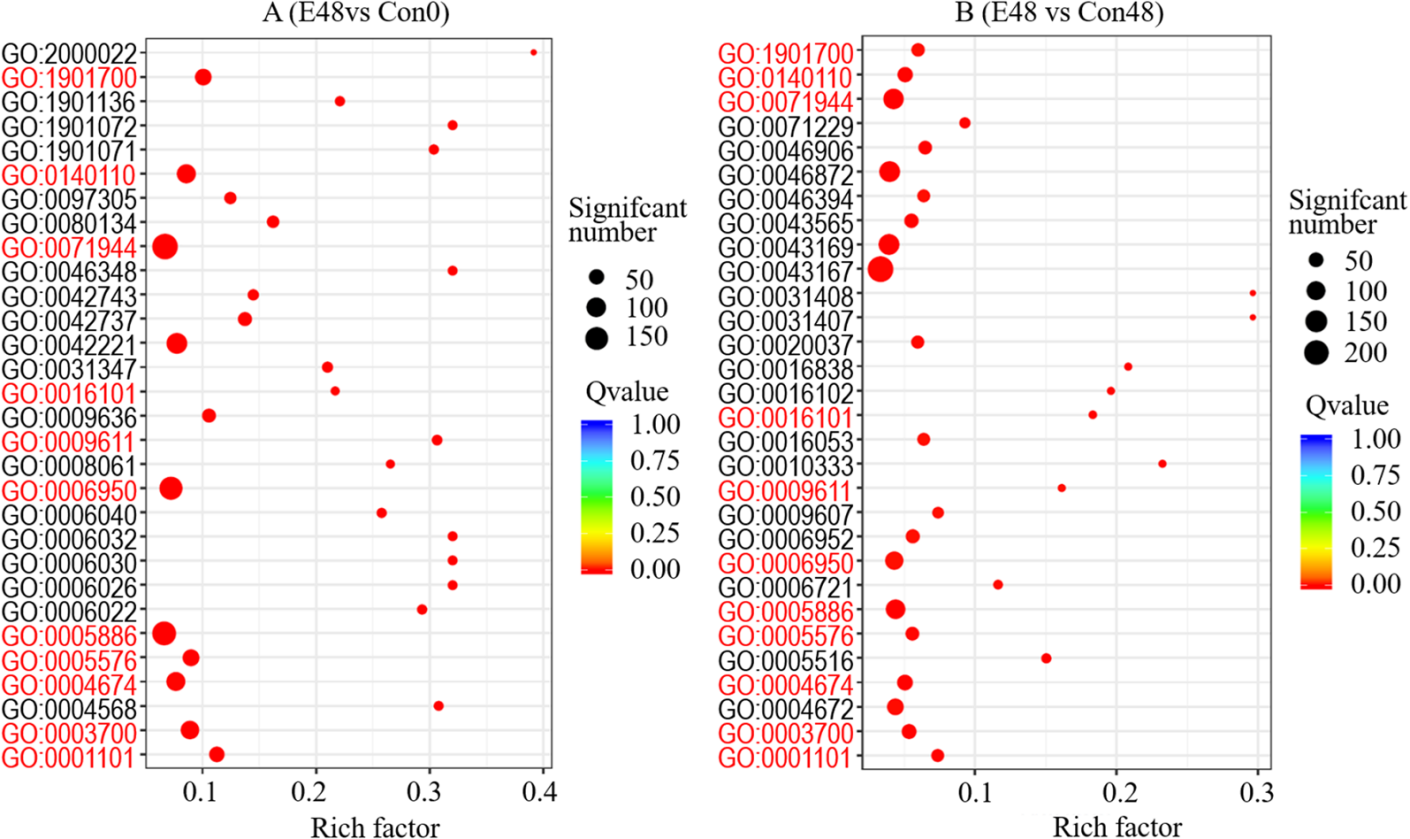
Scatter diagram of GO classification of protein elicitor EsxA-induced differentially transcribed genes in rice

The gene ontology (GO) annotations indicated that EsxA induced the differential transcription (compared with Con0) of genes in 2,612 functional groups. One of the most significantly enriched functional groups was GO: 2000022 [regulation of jasmonic acid (JA)-mediated signaling pathway]. The three enriched functional groups with the most genes were GO: 0071944 (cell periphery), GO: 0005886 (plasma membrane), and GO: 0006950 (response to stress) (Fig. 6A). The comparison with Con48 indicated that the EsxA treatment induced the differential transcription of genes in 2100 functional groups. The most significantly enriched functional groups were GO: 0031408 (oxylipin biosynthetic process) and GO: 0031407 (oxylipin metabolic process). The enriched functional groups with the most differentially transcribed genes were GO: 0043167 (ion binding), GO: 0046872 (metal ion binding), and GO: 0043169 (cation binding) (Fig. 6B). Genes in 11 functional groups were identified as differentially transcribed in both the E48 versus Con0 and E48 versus Con48 comparisons (Fig. 6A, B, classification numbers in red).

#### Functional Analysis of EsxA-induced Up-regulated Genes

**Fig. 7:**
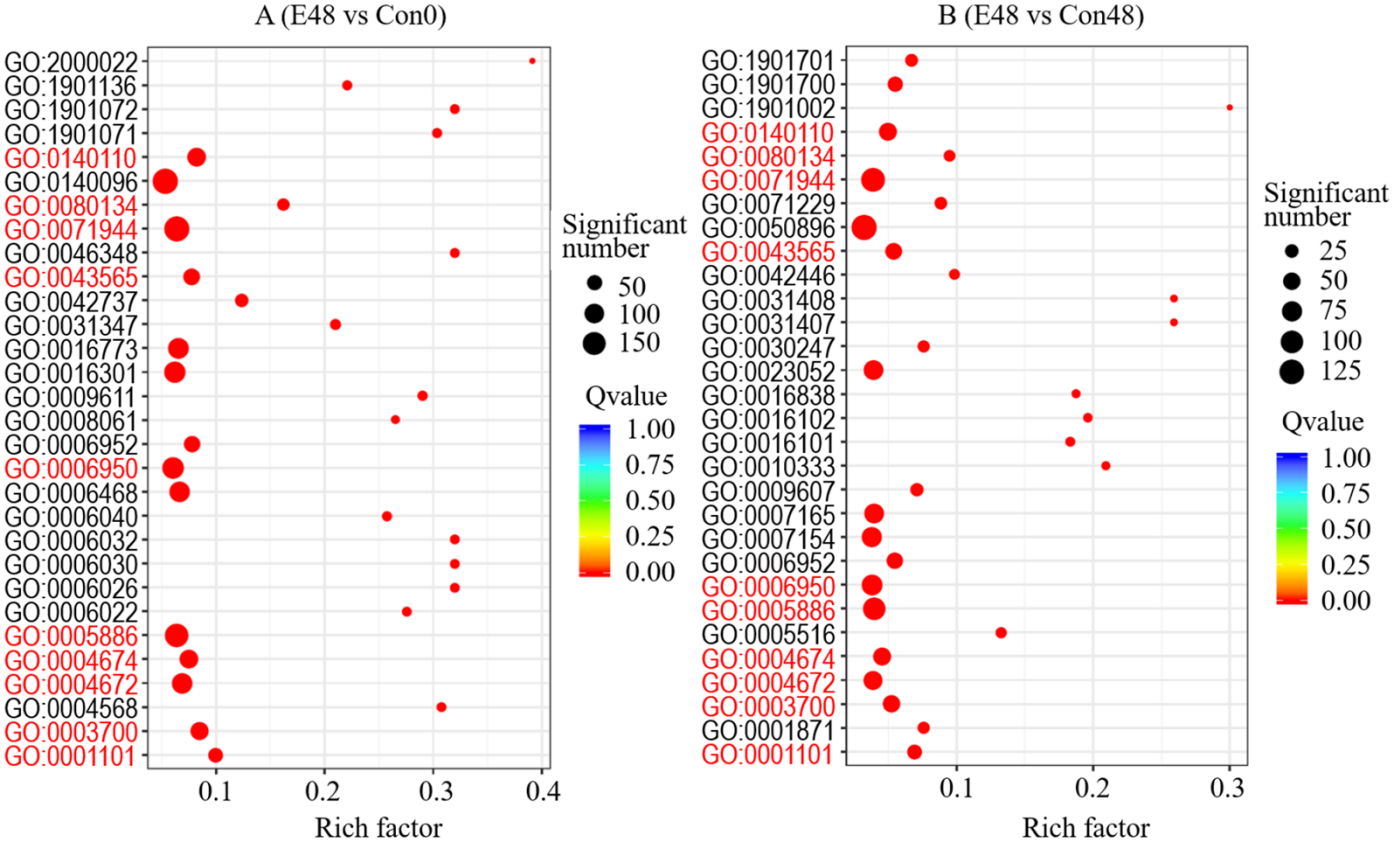
Scatter diagram of GO classification of protein elicitor EsxA-induced up-regulated genes in rice

The GO annotations revealed that EsxA up-regulated the transcription of genes in 2244 functional groups (E48 vs. Con0). One of the most significantly enriched functional groups was GO: 2000022 (regulation of JA-mediated signaling pathway). The four enriched functional groups with the most genes were GO: 0140096 (catalytic activity, acting on a protein), GO: 0005886 (plasma membrane), GO: 0071944 (cell periphery), and GO: 0006950 (response to stress) (Fig. 7A). Compared with Con48, the EsxA treatment up-regulated the transcription of genes in 1807 functional groups. The most significantly enriched functional group was GO: 1901002 (positive regulation of response to salt stress), followed by GO: 0031408 (oxylipin biosynthetic process) and GO: 0031407 (oxylipin metabolic process). The three enriched functional groups with the most genes were GO: 0050896 (response to stimulus), GO: 0071944 (cell periphery), and GO: 0005886 (plasma membrane) (Fig. 7B). Furthermore, genes in 12 functional groups were identified as up-regulated in both the E48 versus Con0 and E48 versus Con48 comparisons (Fig. 7A, B; classification numbers in red).

The genes with EsxA-induced up-regulated transcription levels (E48 vs. Con48) were associated with the cell periphery, plasma membrane, transcription regulator activity, calmodulin binding, signal transduction, defense responses, hormone responses [including abscisic acid (ABA) and JA responses], chitin response, ethanol response, response to oxygen-containing compounds, ABA-activated signaling pathways, JA-mediated signaling pathway regulation, diterpene biosynthesis process, protein phosphorylation, cell communication, and chitinase activity. These functions may contribute to the disease resistance of rice.

#### Disease Resistance-related Genes with EsxA-induced Up-regulated Transcription Levels

The genes with EsxA-induced up-regulated transcription levels included genes related to disease resistance. At 48 h after the EsxA treatment, the transcription levels of genes encoding mitogen-activated protein kinase 2 (MAPK2), NPK1-related PK2, WRKYs, TIFYs, chitinases, PR10, XB15, PODS, PAL, ERF, and MYBS were significantly up-regulated (E48 vs. Con0). The transcription levels of these genes were also detected as up-regulated in the E48 versus Con48 comparison, but the up-regulated transcription level was less than that in the E48 versus Con0 comparison. Additionally, the transcription levels of 12 genes were up-regulated over time in rice that was not treated with EsxA (Con48 vs. Con0); however, the extent of the up-regulation was less than that revealed by the E48 versus Con0 comparison. The PR genes with EsxA-induced up-regulated transcription levels encode the following disease resistance-related proteins: NPK1-related PK, WRKY71, TIFY11A, PR8, chitinase 1, chitinase 3, chitinase 8, root-specific *Oryza sativa* PR10, root-specific rice PR10, root-specific OsPR10a, POD22, POD62, POD22.3, and MYB2 (E48 compared with Con0 and Con48). These results indicated that a few of these genes (12) were up-regulated over time even without EsxA induction (Con48 vs. Con0), while more genes (51) were up-regulated over time only with EsxA induction (E48 vs. Con0), which also up-regulated at the same point in time, compared with control without EsxA treatment (E48 vs. Con48), but no one had higher fold change than that of E48 versus Con0 (Table 1).

**Table 1:** Information regarding disease resistance-related genes with EsxA-induced up-regulated transcription levels

Protein name	Description (gene ID)	Log_2_(Fold Chang) (P < 0.05)			References
		E48 versus Con0	E48 versus Con48	Con48 versus Con0	
MAPK	Multiple stress responsive MAP kinase 2 (Os03g0285800)	3.46	2.68	–	Song and Goodman (2002), Hur and Kim (2014)
MAPKKK	MAPK kinase kinase 6 (Os01g0699500)	3.87	3.64	–	Ma et al. (2017, 2021)
NPK1-related PK	*NPK1-related protein kinase (Os01g0699600)*	5.59	3.56	2.03	Savatin et al. (2014)
ERF	*Ethylene response factor (ERF) 104* (Os08g0474000)	5.79	4.35	–	Pré et al. (2008), Tripathi et al. 2020)
	ERF130 (Os05g0497200)	2.86	1.85	–	
	ERF91 (Os02g0654700)	2.91	1.99	–	
WRKY	WRKY1 (Os01g0246700)	3.16	1.98	–	Yang et al. (2009), Molan and El-Komy (2010)
	WRKY21 (Os01g0821600)	4.73	3.21	–	Zhao et al. (2019) Zhou et al. (2008)
	WRKY24 (Os01g0826400)	4.39	2.89	–	Yokotani et al. (2018)
	WRKY28 (Os06g0649000	4.82	3.72	–	Meng and Wise (2012), Chujo et al. (2013)
	*WRKY45 (Os05g0322900)*	3.52	2.68	–	Shimono et al. (2007), Inoue et al. (2013)
	WRKY53 (Os05g0343400)	2.04	1.29	–	Miao and Zentgraf. (2007), Hu et al. (2012)
	WRKY62 (Os09g0417800)	3.04	2.27	–	Liu et al. (2016)
	WRKY70 (Os05g0474800)	5.10	3.49	–	Hu et al. (2012), Li et al. (2004), Ülker et al. (2007), Knoth et al. (2007)
	WRKY71 (Os02g0181300)	3.85	1.90	1.95	Liu et al. (2007)
	WRKY79 (Os03g0335200)	6.08	4.05	–	Fu et al. (2017)
TIFY	TIFY11A (Os03g0180800)	4.57	2.39	2.18	Ye (2011)
	TIFY11B (Os03g0181100)	3.70	2.74	–	Ye (2011)
	TIFY11E (Os10g0391400)	4.68	4.21	–	Ye et al. (2009)
Chitinase	*PR8, Chitinase 1 (Os10g0416500)*	4.12	2.49	1.62	Schlumbaum et al. (1986), Mourão Filho et al. (2014)
	Chitinase 3 (Os06g0726100)	3.97	1.52	2.44	
	Chitinase 8 (Os10g0542900)	3.50	1.23	2.26	
	Chib3a (Os01g0660200)	3.21	2.28	–	
	Chitinase 11 (Os03g0132900)	2.88	1.97	–	
Glucanase	*Beta-1, 3-glucanase, pathogenesis-related protein 2 (Os01g0940700)*	3.71	2.90	–	Gerhard and Frederick (1999)
	beta-1, 3-glucanase 10 (Os01g0713200)	3.58	2.50	–	
	beta-1, 3-glucanase 11 (Os07g0539100)	2.44	2.15	–	
PR10	Root-specific *Oryza sativa* PR10, PR10a (Os12g0555000)	5.24	3.17	2.08	Takeuchi et al. (2016), Pulla et al. (2010)
	Jasmonate inducible PR10 (Os03g0300400)	2.59	2.00	–	
	PR10B (Os12g0555200)	4.10	3.00	–	
XB15	XA21 binding protein15 (Os03g0821300)	2.27	1.88	–	Park et al. (2008)
AOS	*Allene oxide synthase 1 (Os03g0767000)*	2.39	1.90	–	Gnanaprakash et al. (2013)
	Allene oxide synthase 2 (Os03g0225900)	5.59	2.93	–	Pajerowska-Mukhtar et al. (2009)
Elicitor 5	E3 ubiquitin-protein ligase EL5 (Elicitor 5) (Os02g0559800)	4.84	3.20	–	You et al. (2016), Kumar et al. (2016)
	Elicitor 5 (Os02g0560200)	4.79	3.20	–	
	Elicitor 5 (Os02g0560600)	4.77	3.17	–	
	Elicitor 5 (Os02g0561000)	4.80	3.18	–	
	Elicitor 5 (Os02g0561400)	4.79	3.17	–	
	Elicitor 5 (Os02g0561800)	4.82	3.21	–	
PAL	*Phenylalanine ammonia lyase (Os05g0427400)*	3.33	2.95	–	Tonnessen et al. (2015), Solekha et al. (2020)
POD	Class III peroxidase (POD) 19 (Os01g0787000)	3.96	2.14	1.81	Gao et al. (2010), Wally and Punja. (2010), Takashima et al. (2013)
	*Class III POD22 (Os01g0963000)*	2.54	1.86	–	
	POD22.3 (Os07g0677200)	3.41	1.61	1.79	
	Class III POD59	2.12	1.39	–	
	Class III POD62 (Os04g0688500)	4.20	1.51	2.7	
	Class III POD81 (Os06g0522300)	4.12	1.96	–	
	Class III POD83 (Os06g0521500)	4.49	2.24	–	
Myb	Myb transcription factor2 (Myb2) (Os03g0315400)	3.73	2.09	1.64	Qi (2015)
	Myb4 paralog (Os02g0624300)	6.95	4.10	–	Baldoni et al. (2013)
	Myb4, Myb8 (Os04g0517100)	5.21	3.39	1.82	
NPR	*NPR1-like gene 4 (Os01g0837000)*	2.04	2.01	–	Liu et al. (2005)

**Fig. 8:**
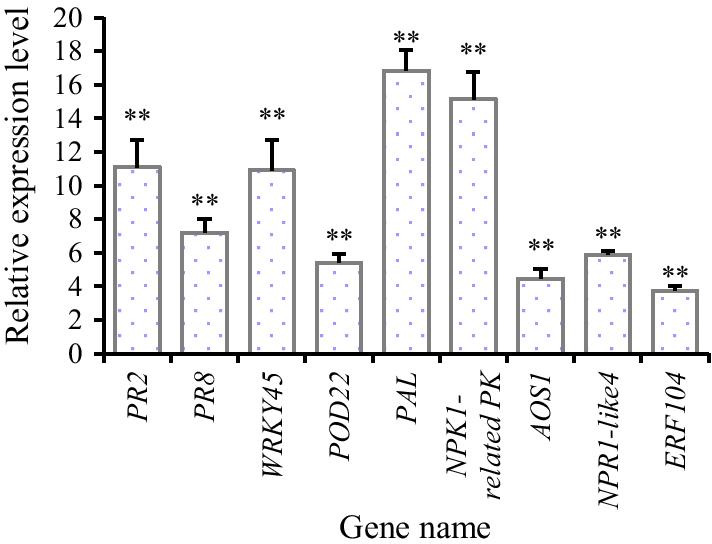
PR genes with EsxA-induced up-regulated transcription levels. The italic font in Table 1 represents the genes used in qPCR validation; “**” above the column indicates the differences between E48 and Con48 were significant (*P* < 0.01)

The italic font represents the genes used in qPCR validation in Fig. 8. “ − ” represents *P* > 0.05

#### Verification of Up-regulated Transcription by qPCR

The qPCR data for the genes encoding PR2, PR8, WRKY45, POD22, PAL, NPK1-related PK, AOS, NPR1-like gene 4, and ethylene response factor 104 (ERF104) were consistent with the transcriptome sequencing results (Fig. 8).

## Discussion

Bases on our studies on EsxA, we believe it a protein elicitor that can trigger ISR in plant, including rice seedling blight and rice blast (Yu et al. 2021a). While other researchers suggest that EsxA can induce immunogenic activity of animal to bacterial diseases (Zhou et al. 2013; Hoang et al. 2013; Yi et al. 2021). Even though there may be differences of diseases that caused by bacterial and fungal pathogens in plant or animal, but the animal’s immunity is similar to plant systemic resistance, they both enhancing organism resistance to some diseases. And we believe that EsxA can trigger ISR in rice based on the transcriptome sequencing results and our prevous study (Yu et al. 2021a).

Plants stimulated by protein elicitors will produce a series of defense responses (e.g., oxidative burst, HR, and nitric oxide accumulation) (Gabriel et al. 2015; Foissner 2000), which subsequently activate the disease resistance-related metabolic activities involving the JA, ethylene, and aABA signaling pathways. Moreover, there is “cross-talk” among the various signaling pathways. For example, the elicitor may interact with JA esters, ethylene signaling pathways, and ROS. This cross-talk integrates multiple signaling pathways and transcription factors (Cheng et al. 2018). The above-mentioned signaling components affect the elicitor signaling network at the transcriptional and metabolic levels to influence plant secondary metabolism (Zhao et al. 2005; Cheplick et al. 2018). The affected secondary metabolites will further regulate plant growth and stress resistance. Most researchs indicates that the elicitor induced resistance is mostly systemic resistance (Wang et al. 2021).

On the basis of the transcriptome sequencing data and the results of our previous studies (Yu et al. 2021b), we speculate that EsxA helps plants establish the first defense response by inducing the ROS burst and HR and by activating the transcription of cell structure-related genes to strengthen the cell wall (e.g., increased PAL levels) (Table 1, Fig. 8). Moreover, EsxA also interacts with membrane receptors, which then activate transcription factors, including WRKYs. These WRKY transcription factors then interact with NPK1-related PK to increase the transcription of downstream defense genes (e.g., PR1, PR2, PR8, and POD genes) (Table 1, Fig. 8) and protein phosphorylation, resulting in the formation of second messengers. Among which, POD activity was confirmed increased in EsxA treated rice plant (Yu et al. 2021b). The amplified signal will induce the ROS burst in other cells via signal transduction pathways. These changes can further regulate salicylic acid-mediated systemic acquired resistance or JA/C_2_H_4_-mediated induced systemic resistance, which ultimately triggers disease resistance-related responses.

Genes encoding EsxA have been commonly detected in the genomes of plant growth-promoting rhizobacteria belonging to the genus *Paenibacillus*, including *P. terrae* NK3-4 (Yu 2019). However, EsxA in *Paenibacillus* species has not been functionally characterized. The results described herein indicate that EsxA regulates the transcription of plant PR genes via molecular interactions to initiate defense-related metabolism, leading to disease resistance. To clarify the mechanism underlying EsxA-induced plant disease resistance and lay the foundation for using EsxA to protect plants from diseases, future studies will be performed to confirmation our hypothesis as the following. First, the molecular interactions between EsxA and host plants at the level of transcription and the post-transcriptional regulation of downstream genes encoding proteins (e.g., PR proteins) that interact with transcription factors should be explored, these interactions increase plant disease resistance through signal transduction pathways that regulate salicylic acid and/or JA defense-related metabolism. Second, EsxA receptor proteins will need to be identified and the binding of EsxA to the receptor protein (or receptor-like protein) should be confirmed in protein interaction analyses. The binding of EsxA to the receptor regulates PR gene transcription or downstream signal transduction to enhance plant disease resistance. Third, an *esxA*-transgenic rice will be constructed and compared with wild varieties to detect the difference of disease resistance, which will also provide materials for rice resistance breeding.

## Conclusions

The EsxA treatment induced seed germination, increased seedling vigor, promoted seedling growth, and enhanced seedling blight resistance in response to the *F. oxysporum* infection. The transcriptome analysis combined with qPCR proved that EsxA induced the differential transcription of rice genes, including the up-regulated transcription of a series of PR genes. These findings may be relevant for the use of EsxA as a protein elicitor to control plant diseases and for the genetic improvement of rice to enhance disease resistance.

## Materials and Methods

### Materials

The *Fusarium oxysporum* strain used in this study was preserved in our laboratory. The cultivated *japonica* rice model species *Oryza sativa* L. was purchased from Kyoto Co., Ltd. Japan. It was used as a typical *japonica* rice genome donor in the International Rice Genome Project, and its genome has been sequenced (Goff 2002). The EsxA exogenously expressed and secreted by *P. pastoris* cells was dissolved in 50 mM PBS and stored at − 80 °C.

### Induction of Rice Seedling Blight Resistance by EsxA

#### Seed-Dipping Treatment

Sterile filter paper was added to six Petri dishes. Rice seeds were soaked in sterile water for 5 days at room temperature and then placed on the filter paper, with 100 seeds per Petri dish. Next, 10 mL EsxA solution (10 μg/mL; prepared in 50 mM PBS, pH 7.5) was added to three Petri dishes, which were then gently shaken to ensure all seeds were fully soaked (marked as EsxA treatment). As the control, a bovine serum albumin (BSA) solution was added to the other three Petri dishes (marked as Con). All samples were incubated at 28 °C for 24 h. Three biological replicate (three Petri dishes) were set for both of the Con and EsxA treatments, respectively.

When the radicles grew out (approximately 1 cm long), 5 mL *F. oxysporum* spore suspension (1 × 10^6^/mL) was added to all six Petri dishes, which were rotated to ensure all radicles were inoculated with spores. seedlings were incubated at 28 °C for 48 h and then at room temperature with a 12-h light:12-h dark cycle.

At 4 and 8 days after the inoculation with *F. oxysporum*, the germination rate was determined by counting the number of sprouted seeds in every Petri dishes. Additionally, the representive seedlings were photographed and the bud length and root length of random 10 seedlings in every Petri dishes were measured. Average germination rate, bud length or root length in each Petri dish was as one of the three biological replicats for both of the Con and EsxA treatments, respecitively. The induction efficiency was calculated using Eq. 1.

1 $${\mathrm{Induction}}\;{\mathrm{efficiency}}\left( \% \right) = \left( {R_{t} - R_{c} } \right)/R_{c} \times 100$$

R_t_: germination rate of EsxA-treated seeds; R_c_: germination rate of control seeds.

#### Seedling-dipping Treatment

Rice seeds were soaked in sterile water for 5 days at room temperature and then placed on sterile filter paper in six Petri dishes, with 16 seeds per Petri dish. To maintain humid conditions, 10 mL distilled water was added to the Petri dishes, which were then incubated at 28 °C for 24 h. When the radicles grew out (approximately 1 cm long), 10 mL EsxA solution (10 μg/mL, prepared in 50 mM PBS, pH 7.5) was added to three Petri dishes (marked as EsxA treatment), which were then gently shaken to ensure all seedlings were fully soaked. As the control, BSA was added to the other three Petri dishes (marked as Con treatment). The seedlings in all Petri dishes were incubated at 28 °C with a 12-h light:12-dark cycle for 48 h. Next, 5 mL *F. oxysporum* spore suspension (1 × 10^6^/mL) was added to all six Petri dishes, which were then rotated to ensure all seedlings were inoculated with spores. Samples were incubated at 28 °C for 48 h and then at room temperature with a 12-h light:12-dark cycle for 5 daysThe bud length and root length of all seedlings in each Petri dishs were measured. Average plant length or root length in each Petri dishes was as one of three biological replicates for both of the Con and EsxA treatments, respectively. The induction efficiency was calculated through germination rate, root length or bud length of seedlings using Eq. 2. Because rice seedling blight pathogen *F. oxysporum* can inject seeds and lead to seed rot, then the seeds can’t germinate, so we calculated induction efficiency using germination rates; it can also inhibit rice buds and roots growing, so we calculated induction efficiency using bud length or root length.

2 $${\mathrm{Induction}}\;{\mathrm{efficiency}}\left( \% \right) = \left( {R_{EsxA} {-}R_{Con} } \right)/R_{Con} \times 100$$

R_t_: rice germination rate, root length or bud length of EsxAtreatment; Rc: rice germination rate, root length or bud length of of Con treatment.

#### Seedling-spraying Treatment

Seedling culture: Rice seeds were soaked in sterile water for 5 days at room temperature and then placed on a sponge mat at the bottom of six plastic pots, with 200 seeds per pot. To maintain humid conditions, the sponge mat was moistened with 10 mL distilled water. Rice seedlings were incubated at 30 °C in darkness for 2 days and then at 25 °C with a 12-h light:12-dark cycle for 48 h (plant length was approximately 1.5 cm and root length was approximately 2.5 cm). Rice seedlings were sprayed with sterile water to maintain humid conditions for growth.

EsxA spray treatment and analysis of plant biological indices: Seedlings in three pots were sprayed with 20 μg/mL EsxA solution, with 1 mL per pot (marked as EsxA treatment). As the control, the seedlings in the other three pots were sprayed with a BSA solution (marked as Con treatment). After a 48-h incubation, the remaining EsxA and BSA solutions in the pots were discarded, after which 10 mL sterile water was added to the pots.

Inoculation with *F. oxysporum* and analysis of biological indices: 48 h after EsxA treatment, seedlings in each of the six pots were dipped in 10 mL *F. oxysporum* spore suspension (1 × 10^6^/mL) for 5 min. After removing excess spore suspension from the pots, 10 mL sterile water was added to each pot to keep humidity for rice seedlings growth. Following a 7-day incubation at room temperature (15–20 °C) under natural light, 20 seedlings were random sampled from each pot and analyzed (i.e., plant length, number of roots, root length, and number of white roots). Average plant length, number of roots, root length, or number of white roots of the 20 seedlings in each pot was as one of three biological replicates for both of the Con and EsxA treatments, respectively. The rest seedlings in pots were contined incubating for 7 days for the following analysis.

Analysis of the effect of EsxA on rice seedling blight resistance: At 14 days after the inoculation with *F. oxysporum*, all of the rice plants in each pot were examined, the number of rice seedlings exhibiting sheath rot and stem rot symptoms was recorded. Disease incidence of seedling blight which leaded to sheath rot and stem rot, and the EsxA induction efficency were analyzed using Eqs. 3 and 4. For the caculation of disease incidence of seedling blight, all of the remained seedlings in each pot were observed to determin the the incidence rate of sheath rot and stem rot, and disease incidence of total 180 seedlings in each pot was as one of the three biological replicates.

3 $${\mathrm{Disease}}\;{\mathrm{incidence}}\left( \% \right) = \left( {{\mathrm{number}}\;{\mathrm{of}}\;{\mathrm{infected}}\;{\mathrm{plants}}/{\mathrm{total}}\;{\mathrm{number}}\;{\mathrm{of}}\;{\mathrm{plants}}} \right) \times 100$$

4 $${\mathrm{Induction}}\;{\mathrm{efficiency}}(\% ) = \left( {{\mathrm{disease}}\;{\mathrm{incidence}}\;{\mathrm{of}}\;{\mathrm{Con}} - {\mathrm{disease}}\;{\mathrm{incidence}}\;{\mathrm{of}}\;{\mathrm{EsxA}}} \right)/{\mathrm{disease}}\;{\mathrm{incidence}}\;{\mathrm{of}}\;{\mathrm{Con}}$$

### Analysis of Rice Transcriptome

#### RiceTreatment and Sampling for Transcriptome-sequencing Analysis

Rice seeds were treated and seedlings were cultured as described in the above section. Seeds were sawn in nine pots. After emrgenced, rice seedlings were incubated in a greenhouse with a 12-h light (28 °C): 12-dark (22 °C) cycle for 7 days to produce enough biomass for sample analysis. At first, as the control, rice seedlings in each of the three pots were collected, respectively. Then the roots were cut off followed by being washed with distilled water, dried, and wrapped in tinfoil then quickly frozen in liquid nitrogen, plants (approximate 1 g) collected from each pot was as one of the three biological replicate, respectively (marked as Con0). The frozen samples were stored at − 80 °C. At the same time, the seedlings in other three pots were sprayed with 20 μg/mL EsxA solution, with 1 mL per pot (marked as E48 treatment). As another control for that of 48 h after the EsxA treatment, the seedlings in the last three pots were sprayed with a BSA solution (marked as Con48). After 48 h, seedlings were sampled randomly in each of the six pots (the three pots marked E48, and the another three pots marked with Con48), the roots of rice seedlings in each pot were cut off, and then the rice plants (approximate 1 g) were washed with distilled water, dried, and wrapped in tinfoil, then quickly frozen in liquid nitrogen, plants sampled from each pot as one of the three biological replicates of E48 and Con0 treatments. The total nine samples (three biological replicates for each of the Con0, Con48 and E48 treatment) were sent to Bioengineering (Shanghai) Co., Ltd. in dry ice for a transcriptome sequencing analysis according a transcriptome sequencing projects with the reference genome for rice (*Oryza sativa* L.).

We extracted RNA from young seedlings that including sheaths and leaves but excluding roots, because we predicted that EsxA may induce systemic resistance in plant based on our previous studies and references, and to investigate whether EsxA can induce systemic resistance to seedling blight, but not the local resistance in roots.

The purpose of comparing E48 versus Con0 was to investigate which of the genes differentially expressed in rice induced by EsxA, were also differentially expressed without EsxA induction, but only changed over time (Con48 vs. Con0), and the extent to which they differentially express themselves, as well as which genes do not change their expression levels over time and are differentially expressed only when induced by EsxA.

#### Transcriptome-sequencing and Analysis

Rice total mRNA of the nine samples were isolated using E.Z.N.A.® Total RNA Kit (Qmega Bio-tek, Inc., GA, USA) according to the manufacturer’s instructions, followd by RNase-free DNaseI treating to remove possible residual DNA; then mRNA library was constructed as the following processes: the mRNA fragmentation, double stranded cDNA synthesis, chemical modification of cDNA fragments, magnetic bead purification and fragmentation sorting, library amplification, detection and quality control, and the sequencing by Illumina Hiseq™ were performed successively.

After data evaluation and quality control using FastQC, short reads were mapped to the rice genome and annotated gene; sequences mapped to the genome were assembled using StringTie and then compared with known gene models using GFFCompare (version 0.10.1) to discover new transcription regions.

Expression level analysis was conducted usig StringTie (version 1.3.3b), and calculated -log_2_(Fold Change), and expression difference analysis was performed using DESeq2 (version 1.12.4) in R Package and expression difference was statistic analyzed using DESeq (qValue < 0.05, and |FoldChange|> 2). Volcano map was drawed based on the gene expression levels between treatmtments; Principal component analysis (PCA) was performed based on the gene expression level among treatmtments using vegan in R Package; and gene GO enrichment analysis was performed using topGO (version 2.24.0 in R Package), and scatter diagram of differentially expressed genes between treatments was maped based GO classification results using ggplot2 in R package.

### Verification of Up-regulated Genes in Rice by qPCR

#### Primer Design

Nine genes with up-regulated transcription revealed by the transcriptome sequencing data were selected, which had been reported as PR genes that can induce systemic resistance to diseases. The genes were analyzed by qPCR to check the transcriptome sequencing results were reliable, with the 18S rRNA gene used as the internal reference control. Total RNA was extracted from rice samples using the E.Z.N.A.^®^ Total RNA Kit (Qmega Bio-tek, Inc., Shanghai, China). The primers used in this study are listed in Table 2.

**Table 2:** Primer details

Gene name	Gene ID	Primer sequences
*PR2*	Os01g0940700	F:5′-ATTGGTCCTTGGAGTTGCG-3′
		R:5′-GATGCCGTTGGACTTGTAGAG-3′
*PR8*	Os10g0416500	F:5′-CAGCTACAAGTTTGAGTACGAGACC-3′
		R:5′-CCAATCGGCACATAAGTCCA-3′
*WRKY45*	Os05g0322900	Fn:5′-ATCTGGACGACATTATGGGTTT-3′
		Rn:5′-GAGACGACACATCAACAAGGAAT-3′
*POD22*	Os01g0963000	F:5′-TGCTGAACACCAACGACAC-3′
		R:5′-CCATCTTGACGACGGAGTAGA-3′
*PAL*	Os05g0427400	F:5′-GACGGCAGGAAGGTGAAC-3′
		R:5′-GGTGAGGTGGTCGGTGTA-3′
*NPK1-related PK*	Os01g0699600	F:5′-ATGCTTCACAAGGAACCCAA-3′
		R:5′-GCCTTCTTCTACTTCGTCGTCT-3′
*AOS1*	Os03g0767000	F:5′-GCTGGTGAAGAAGGACTACGA-3′
		R:5′-CCGCCGAACGAGTTGAAG-3′
*NPR1-like5*	Os01g0837000	F:5′-GAGACCACAAGACTGCGTATGA-3′
		R:5′-CTGAGTTCCTTAGCAATCCCA-3′
*ERF104*	Os08g0474000	Fn:5′-ATGGGAGGCAACCAGGAGTA-3′
		Rn:5′-GAGATGACATGGAGCAGCGT-3′
*18S rRNA*		F:5′-CATAAACGATGCCGACCAG-3′
		R:5′-CACCACCCATAGAATCAAGAAA-3′

#### cDNA Synthesis

After analyzing the extracted RNA by agarose gel electrophoresis, the RNA quality and concentration were determined using the SMA4000 microspectrophotometer [Merinton (Beijing) Instrument Co., Ltd., Beijing, China]. The high-quality RNA was reverse transcribed to synthesize cDNA. Briefly, 1500 ng total RNA was added to a nuclease-free PCR tube in an ice bath, after which 1 μL Random Primer P (DN)_6_ (100 pmol), 1 μL dNTP Mix (0.5 mM final concentration), and RNase-free ddH_2_O was added for a final volume of 14.5 μL. After gently mixing, the reaction mixture was centrifuged for 3–5 s. It was then incubated at 65 °C for 5 min and then in an ice bath for 2 min before centrifuging again for 3–5 s. Next, 4 μL 5 × RT Buffer, 0.5 μL Ribolock RNase Inhibitor (20 U) (Thermo Scientific), and 1 μL Maxima Reverse Transcriptase (200 U) were added to the PCR tube, which was then gently mixed and centrifuged for 3–5 s. The RNA was reverse transcribed to cDNA in a thermal cycler under the following conditions: 25 °C for 10 min, 50 °C for 30 min, and 85 °C for 5 min. The PCR tube was then stored at − 20 °C.

#### qPCR Analysis

The cDNA samples were diluted 20 times and used as the template for a qPCR, which was completed using the StepOne Plus system (ABI, Foster, CA, USA) and the 2 × SG Fast qPCR Master Mix. The 20 μL reaction mixture included 10 μL SG Fast qPCR Master Mix, 0.4 μL F primer (10 μM), 0.4 μL R primer (10 μM), 7.2 μL ddH_2_O, and 2 μL cDNA template. The qPCR program was as follows: 95 °C for 3 min; 45 cycles of 95 °C for 5 s and 60 °C for 30 s; dissociation according to instrument guidelines. The qPCR analysis was repeated three times.

### Data Analysis

The SPSS 13.0 software (Chicago, USA) was used to evaluate the significance of the difference between two variables. A One-way ANOVA was performed for independent replicates of the same trial. The significant difference was determined at 0.05 levels and 0.01 levels. Paired-samples T test was conducted to detect whether the data violate the assumption. Bar graphs were prepared using Excel 2010.

## Supplementary Information

**Additional file 1**. E48 vs Con0 and E48 vs Con48, which contains 1,376 differential transcribed genes between E48 and Con0, as well as 771 differential transcribed genes between E48 and Con48 with gene ID description, log_2_ Fold change and protein name.

## Data Availability

All data generated or analyzed during this study are included in this article and its Additional file 1.

## Abbreviations

ABA: Abscisic acid
AOS: Allene oxide synthase
Elicitor 5: E3 ubiquitin-protein ligase EL5
HR: Hypersensitive response
ERF: Ethylene response factor
GO: Gene ontology
JA: Jasmonic acid
ISR: Induced systemic resistance
MAPK: Mitogen-activated protein kinase
MAPKKK: MAPK kinase kinase
MYB: V-myb avian myeloblastosis viral oncogene homolog
NPK: Nicotiana protein kinase
NPR: NPR1-like gene
PAL: Phenylalanine ammonia lyase
PK: Protein kinase
POD: Peroxidase
PR gene: Pathogenesis-related gene
qPCR: Quantitative real-time PCR
ROS: Reactive oxygen species
TIFY: Threonine-isoleucine-phenylalanine-tyrosin motif
WRKY: Transcriptor containing Tryptophan-Arginine-Lysine-Tyrosin conservative domain
XB: Xa21 binding protein
